# Reduced sleep duration mediates decreases in striatal D2/D3 receptor availability in cocaine abusers

**DOI:** 10.1038/tp.2016.14

**Published:** 2016-03-08

**Authors:** C E Wiers, E Shumay, E Cabrera, E Shokri-Kojori, T E Gladwin, E Skarda, S I Cunningham, S W Kim, T C Wong, D Tomasi, G-J Wang, N D Volkow

**Affiliations:** 1 National Institute on Alcohol Abuse and Alcoholism, Laboratory of Neuroimaging, National Institutes of Health, Bethesda, MD, USA; 2 Addiction Development and Psychopathology Lab, Department of Psychology, University of Amsterdam, Amsterdam, The Netherlands; 3 Research Centre—Military Mental Health, Ministry of Defense, Utrecht, The Netherlands; 4 National Institute on Drug Abuse, National Institutes of Health, Bethesda, MD, USA

## Abstract

Neuroimaging studies have documented reduced striatal dopamine D2/D3 receptor (D2/D3R) availability in cocaine abusers, which has been associated with impaired prefrontal activity and vulnerability for relapse. However, the mechanism(s) underlying the decreases in D2/D3R remain poorly understood. Recent studies have shown that sleep deprivation is associated with a downregulation of striatal D2/D3R in healthy volunteers. As cocaine abusers have disrupted sleep patterns, here we investigated whether reduced sleep duration mediates the relationship between cocaine abuse and low striatal D2/D3R availability. We used positron emission tomography with [^11^C]raclopride to measure striatal D2/D3R availability in 24 active cocaine abusers and 21 matched healthy controls, and interviewed them about their daily sleep patterns. Compared with controls, cocaine abusers had shorter sleep duration, went to bed later and reported longer periods of sleep disturbances. In addition, cocaine abusers had reduced striatal D2/D3R availability. Sleep duration predicted striatal D2/D3R availability and statistically mediated the relationship between cocaine abuse and striatal D2/D3R availability. These findings suggest that impaired sleep patterns contribute to the low striatal D2/D3R availability in cocaine abusers. As sleep impairments are similarly observed in other types of substance abusers (for example, alcohol and methamphetamine), this mechanism may also underlie reductions in D2/D3R availability in these groups. The current findings have clinical implications suggesting that interventions to improve sleep patterns in cocaine abusers undergoing detoxification might be beneficial in improving their clinical outcomes.

## Introduction

Cocaine abusers when compared with healthy non-drug abusing controls have reduced striatal dopamine D2/D3 receptor (D2/D3R) availability whether they are tested during early withdrawal or after protracted detoxification reviewed in refs 1, 2, and these reductions persist after months of abstinence.^3^ Reduced striatal D2/D3R availability in cocaine abusers has been associated with increased drug craving,^1^ risk of relapse,^4^ impaired activity in prefrontal brain regions involved with self control,^3^ future cocaine preferences^5^ and poor treatment outcomes.^6^ In preclinical studies, low D2/D3R are associated with a propensity to administer higher doses of cocaine in nonhuman primates^7^ and with compulsive patterns of cocaine intake in rats.^8^ As such, reduced striatal D2/D3R may motivate individuals to use drugs of abuse to increase dopaminergic neurotransmission as a way of self-medication. In nonhuman primates, continuous cocaine administration was shown to reduce D2/D3R availability.^7^ These reductions remained constant following a year of cocaine administration and persisted after a year of abstinence.^7^ Although cocaine-induced increases in dopamine may influence D2/D3R receptor expression directly, the exact mechanism(s) underlying the decrease in D2/D3R associated with chronic cocaine exposures remain poorly understood.

Acute sleep deprivation has been shown to decrease striatal D2/D3R availability in healthy volunteers, which correlated with worse performance on visual attention tasks.^9, 10, 11^ These reductions in D2/D3R were initially interpreted to reflect increased dopamine release (interfering with the binding of the radiotracer). However, a subsequent imaging study that used methylphenidate as a challenge in humans along with microdialysis studies in rodents showed no evidence of increased dopamine release with sleep deprivation, thus indicating downregulation of D2/D3R receptors.^9^

Cocaine use disorders are associated with significant sleep disturbances.^12^ Acute cocaine abuse increases wakefulness and reduces sleep duration^13^ and chronic cocaine abusers suffer from a decrease in sleep quality and duration,^14, 15^ which has also been described in rodents that self-administered cocaine.^16^ Delays in sleep onset have been shown,^14^ which progressively worsened over the course of abstinence.^17^ Moreover, Shumay *et al.*^18^ showed that a variant in the *PER2* gene, which is involved in the regulation of circadian rhythms, was associated with lower striatal D2/D3 availability and was more prevalent in cocaine abusers than controls; this suggests a relationship between sleep rhythms, cocaine abuse and striatal D2/D3Rs. These findings together led us to hypothesize that sleep disturbances contribute to reductions in striatal D2/D3R availability in cocaine abusers.

To test this hypothesis, we measured sleep quality and striatal D2/D3R availability in a group of 24 active cocaine abusers and 21 healthy controls using positron emission tomography (PET) with [^11^C]raclopride. Participants in both the groups were matched for age, sex, education, body mass index, handedness and ethnicity. We hypothesized that cocaine abusers would have reduced sleep durations and reduced striatal D2/D3R availability compared with controls. We also hypothesized that sleep duration would mediate the reductions in striatal D2/D3R.

## Materials and methods

### Participants

Data of 24 cocaine abusers (two females) and 21 controls (one female) were acquired from studies at Brookhaven National Laboratory, for which [^11^C]raclopride scans and sleep data were available.^19, 20^

Groups were matched for age, education, Wechsler adult intelligence scale IQ score^21^ (see Table 1), gender (*χ*^2^=0.23, *P*=0.63), ethnicity (cocaine: 20 African American, three Caucasian, one Hispanic; controls: 14 African American, five Caucasian, one Hispanic, one mixed ethnicity; *χ*^2^=2.37, *P*=0.50) and handedness (three left-handers in both groups, *χ*^2^=0.03, *P*=0.86). There were more smokers among cocaine abusers (20 smokers, four non-smokers) than in the control group (two smokers, one ex-smoker, 18 non-smokers; *χ*^2^=24.5, *P*<0.0001). Despite a previous report that found lower prevalence of caffeine use in cocaine abusers than in a non-abusing population,^22^ our data show no differences in caffeine use between cocaine abusers (8/24) and controls (6/21) group (*χ*^2^=0.12 *P*=0.73).

The participants were screened with a detailed medical history, physical and neurological examinations, EKG, breath CO, routine blood tests and urinalysis, and urine toxicology for psychotropic drugs to ensure that they fulfilled inclusion and exclusion criteria. Cocaine abusers (all outpatients) fulfilled DSM-IV criteria for current cocaine dependence, were active users for at least the prior 6 months (smoking free-base or crack, at least ‘4 grams' a week) and were intend to continue to use cocaine at the time of testing.^19, 20^ Exclusion criteria for all the participants included current or past psychiatric disease (other than cocaine dependence in the cocaine group); past or present history of neurological, cardiovascular or endocrinological disease; history of head trauma with loss of consciousness greater than 30 min; and current medical illness and drug dependence other than nicotine (and cocaine in the cocaine group). The control group was free of psychoactive medications in the past month. Table 1 provides demographic and clinical information on the two groups. Written informed consent was obtained from the participants before inclusion in the study, as approved by the Committee on Research Involving Human Subjects, Stony Brook University and the Radioactive Drug Research Committee, Brookhaven National Laboratory.

### Sleep measures and questionnaires

The participants were asked to rate their typical daily sleep quality (that is, total hours of sleep per night, bedtime, wake up time, duration of wake periods, feeling of rested in the morning (yes/no)). A licensed psychiatric nurse and sleep researcher performed these interviews. If subjects reported being sleep deprived on the day of the PET scan, the subjects were rescheduled for a different day on which they slept according to their typical sleep times. Further behavioral assessments included a drug history form on smoking and cocaine use, the Beck's Depression Inventory^23^ and the subscales inattention, hyperactivity and impulsivity of the Conners Adult ADHD Rating Scale (CAARS) long version.^24^

### PET imaging, processing and analyses

The [^11^C]raclopride scans were performed on a Siemens, HR+ scanner (resolution 4.5 × 4.5 × 4.5 mm full-width half-maximum, 63 slices) at the BNL Brain Imaging Center. Subjects were abstinent from cigarette smoking and caffeine at least 2 h before scanning to reduce the influence of direct nicotine and caffeine exposure on [^11^C]raclopride binding. The procedures for subjects positioning, scanning protocols and data processing have been described previously.^3, 19^ In short, emission scans were started immediately after injection of 4–8 mCi (specific activity 0.5–1.5 Ci μm^−1^ at the end of bombardment). Twenty dynamic emission scans were obtained from the time of injection up to 54 min and arterial sampling was used to quantify total carbon-11 and unchanged [^11^C]raclopride in plasma. The distribution volume, corresponding to the ratio of the radiotracer's tissue concentration to that of its plasma concentration, was estimated for each voxel using a graphical analysis technique for reversible systems that does not require blood sampling.^25^ These images were spatially normalized to the Montreal Neurological Institute (MNI) stereotactic space using Statistical Parametric Mapping (SPM) version 8 and re-sliced using 2-mm isotropic voxels. A previously developed custom MNI template using distribution volume images from 34 healthy subjects,^26^ was used for normalization. Distribution volume ratios, which correspond to the non-displaceable binding potential in each voxel, were obtained by normalizing the intensity of the distribution volume images to that in the cerebellum (left and right regions of interest).

For the second level analyses, we calculated the following contrasts in SPM8: (1) the effect of group on D2/D3R using a two-sample *t*-test and (2) the effect of sleep on D2/D3R while correcting for group using a one-sample *t*-test with sleep duration as regressor of interest and group as regressor of no interest. As the striatum has the highest density of D2/D3R, for which [^11^C]raclopride is a high-affinity ligand, we masked these second level analyses with a [^11^C]raclopride striatal mask.^26^ This bilateral mask, which has a volume of 50 ml (6228 voxels with 2-mm isotropic resolution) and includes dorsal and ventral caudate, putamen and globus pallidus, reflects high [^11^C]raclopride binding and was used for small volume correction of the results in SPM8, with a significance threshold of *P*<0.005 uncorrected (*P*_u_) and cluster threshold of 100 voxels. We also report the family-wise error (FWE)-corrected results at cluster level (*P*_FWE_) using random field theory.

We were further interested in whether the duration of sleep mediated the effect of group on striatal D2/D3R availability. For this purpose, we calculated regional Bmax/KD values for hand-drawn caudate, putamen and ventral striatum (VS) using a procedure previously described.^27^ Manually delineated regions of interest had the same size and shape across subjects. The ratio of the distribution volume in striatal regions was computed to that in the cerebellum to obtain the non-displaceable binding potential, which was used as a quantification of D2/D3R availability.^28^

### Mediation analyses

**Figure 1:**
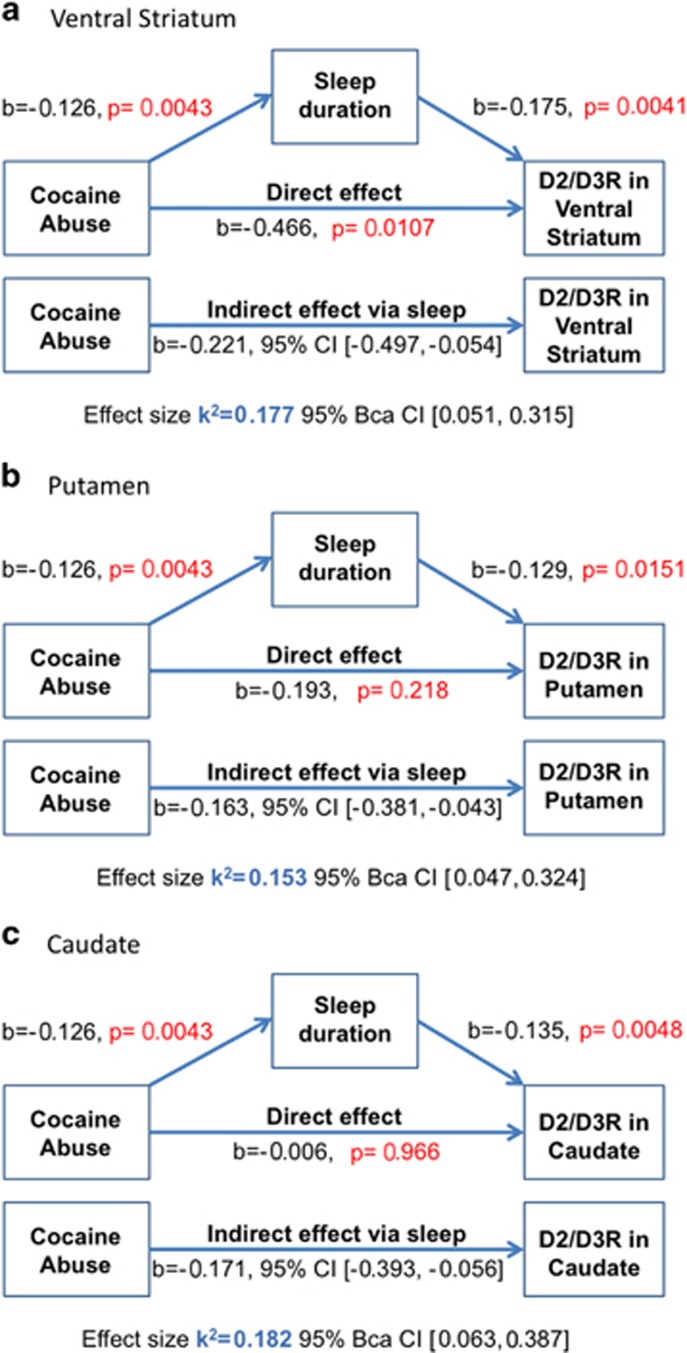
The estimation of the direct and indirect effect of cocaine abuse on striatal D2/D3R (Bootstrap resamples: 1000) in the (**a**) VS, (**b**) putamen and (**c**) caudate. Cocaine abuse was a predictor of sleep duration (*P*<0.005), and sleep duration was a predictor of D2/D3R in VS (*P*<0.005), putamen (*P*<0.05) and caudate (*P*<0.005). Bootstrapping analyses showed that the relationship between cocaine abuse and reduced D2/D3R availability in VS, putamen and caudate was statistically mediated by sleep duration (95% CI≠0). CI, confidence interval; VS, ventral striatum.

We tested the indirect effect of cocaine abuse on striatal D2/D3R mediated by sleep duration using PROCESS bootstrapping and bias-corrected 95% confidence intervals (CIs)^29^ for SPSS 22 (IBM, Armonk, NY, USA). With this software, we calculated the total effect of predictor cocaine abuse (categorical variable cocaine abuse=1, healthy control=0) on outcome striatal D2/D3R, as well as the direct effect of cocaine abuse on striatal D2/D3R in the presence of the mediator sleep duration (see Figure 1). We further calculated the effect of cocaine abuse on the mediator sleep duration; the effect of cocaine abuse on outcome striatal D2/D3R via sleep duration (that is, indirect effect); and the effect of sleep duration on striatal D2/D3R in the presence of cocaine abuse.^30^ Figure 1 provides an overview of the model, showing the direct effect of cocaine abuse on striatal D2/D3R as well as its indirect effect via sleep.

The primary interest for mediation analyses was the ventral striatum, in which sleep has been shown to decrease D2/D3R binding.^9^ We also ran the analysis separately for the putamen and caudate.

To explore potential covariates, we did the following: (1) correlated age, body mass index, gender, caffeine intake and cigarette pack-years with cocaine abuse, D2/D3R and sleep duration, (2) performed an analysis of variance to evaluate the effect of ethnicity on D2/D3R and sleep duration (dependent variables: D2/D3R and sleep duration) and (3) performed a *χ*^2^-test to test potential group differences in ethnicity. Besides a significant regression between cigarette pack-years and group (that is, cocaine abusers smoked more than controls), none of the variables correlated with cocaine abuse, striatal D2/D3R or sleep duration (all *P*-values >0.05, see Table 2 for zero-order correlation coefficients). That is, despite previous findings,^9^ age did not predict D2/D3R in any striatal area (*P*>0.140; see Table 2), which might reflect the restricted age range of our participants.

## Results

### Sleep measures in cocaine abusers versus controls

Cocaine abusers had shorter overall sleep durations, went to bed later (but did not wake up earlier), and reported a longer total duration of wake periods during the night (see Table 1 for statistics). However, there were no differences in feeling rested after sleep (cocaine: 19 yes/5 no, healthy controls: 18 yes/3 no *χ*^2^=0.33, *P*=0.57).

The mediation model showed that the effect of cocaine abuse on the mediator sleep duration was significant for all the three regions of interest (*b*=−1.26, *t*=−3.01, *P*=0.0043; see Figure 1).

### Effect of cocaine abuse on striatal D2/D3R availability

Without sleep in the model, group significantly predicted D2/D3R availability in the VS (*b*=−0.687, *t*=−4.05, *P*=0.0002) and putamen (*b*=−356, *t*=−2.40, *P*=0.021), but not in the caudate (*b*=−0.165, *t*=−1.22, *P*=0.23; see Table 2 for zero-order correlations between these variables). This constitutes the total effect of cocaine abuse on striatal D2/D3R, where the total effect is defined as the sum of the indirect and direct effect of cocaine abuse on striatal D2/D3R that are shown in Figure 1.

**Figure 2:**
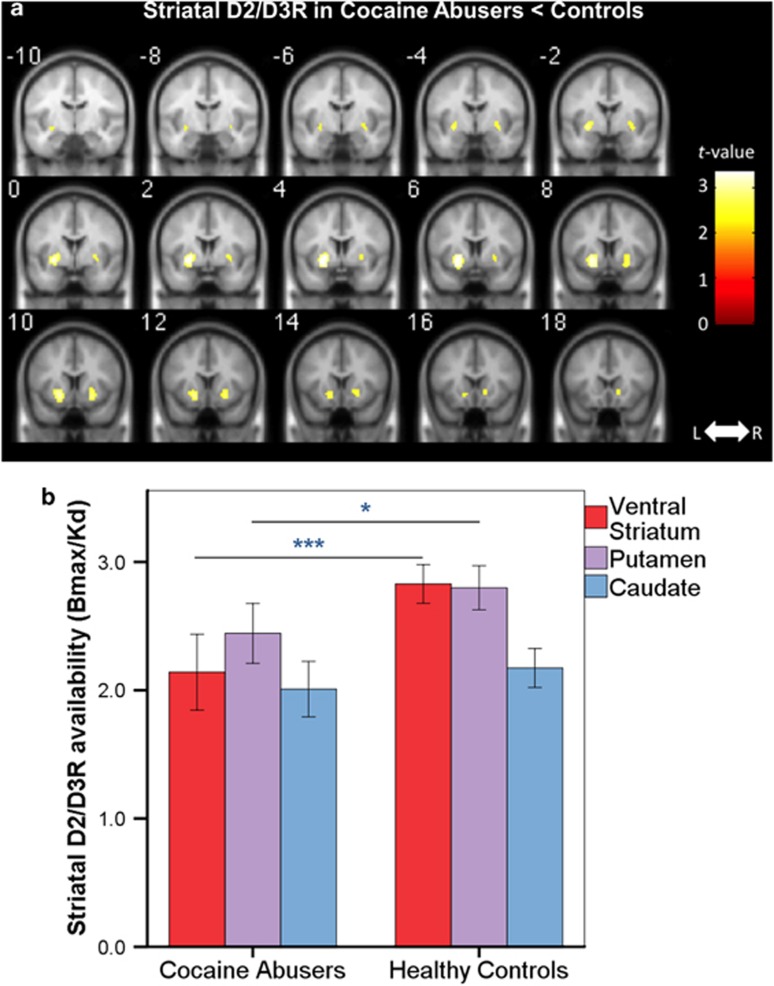
(**a**) Cocaine abusers had decreased striatal D2/D3R availability compared with control subjects (peak left=[−26, 4, −6], *k*=290, *t*=3.37, *P*_u_=0.001, *P*_FWE_=0.041; peak right=[24, 10, −10], *P*=0.003, *k*=22, *t*=2.89, *P*_u_=0.003). For visualization purposes, the SPM threshold was set at *P*_u_<0.01. (**b**) ROI analyses showed that cocaine abusers had lower D2/D3R availability in VS (*P*<0.0001) and putamen (*P*=0.021), but not caudate (*P*=0.23), compared with healthy controls. L, left; R, right; ROI, region of interest; SPM, Statistical Parametric Mapping.

We verified these results using SPM and found decreased D2/D3R availability in cocaine abusers versus controls in the striatum (peak left MNI [x, y, z]=[−26, 4, −6], *k*=290, *t*=3.37, *P*_u_=0.001, *P*_FWE_=0.041 (cluster level); and a smaller cluster in the right striatum=[24, 10, −10], *P*=0.003, *k*=22, *t*=2.89, *P*_u_=0.003; Figure 2), covering the VS and putamen.

### Effects of sleep duration on striatal D2/D3R availability

Hours of sleep predicted D2/D3R availability in all striatal regions of interest (all *P*<0.002; see Table 2 for correlation coefficients).

Mediation analyses showed that these effects also held when including group in the model (VS: *b*=0.175, *t*=2.58, *P*=0.0134; putamen: *b*=0.129, *t*=2.54, *P*=0.015; caudate: *b*=0.135, *t*=3.27, *P*=0.002; see Figure 1).

**Figure 3:**
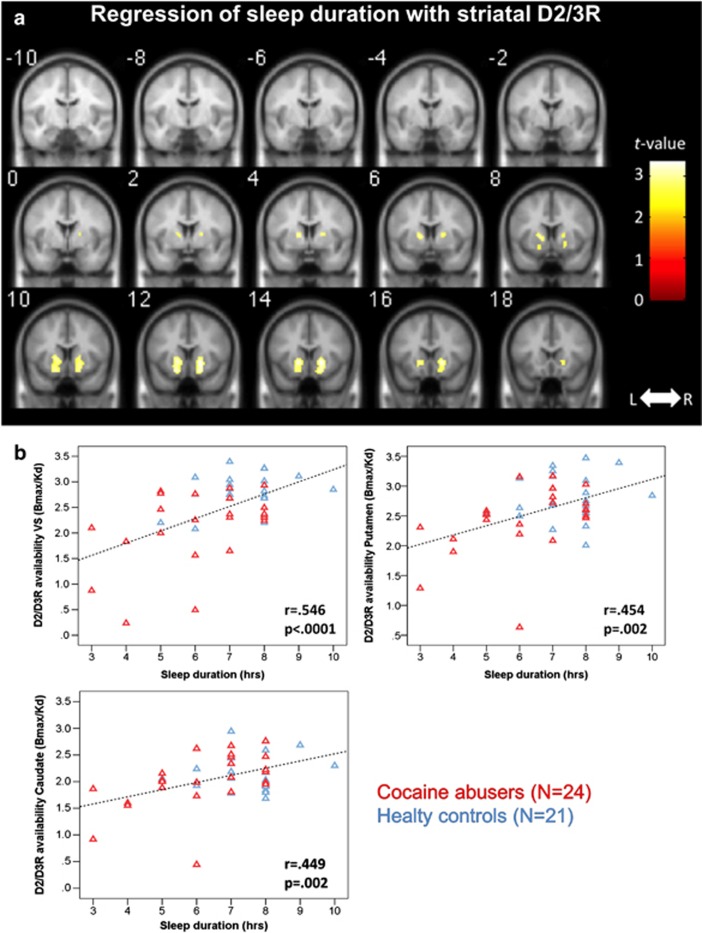
(**a**) Sleep durations predicted bilateral striatal D2/D3R availability, corrected for group (peak left=[−16, 12, −2], *k*=117, *t*=3.02, *P*_u_=0.002; peak right=[18, 14, −4], *k*=106, *t*=3.17, *P*_u_=0.001). For visualization purposes, the SPM threshold was set at *P*_u_<0.01. (**b**) Regression slopes of sleep duration with D2/D3R availability in VS, caudate and putamen in both the groups pooled together. L, left; R, right; SPM, Statistical Parametric Mapping; VS, ventral striatum.

We verified these results in SPM and found a bilateral effect of sleep duration on striatal D2/D3R, corrected for group (peak left=[−16, 12, −2], *k*=117, *t*=3.02, *P*_u_=0.002; peak right=[18, 14, −4], *k*=106, *t*=3.17, *P*_u_=0.001; see Figure 3) covering VS, putamen and caudate.

### Mediation analyses of sleep and striatal D2/D3R

When including sleep as a mediator, there was a significant indirect effect of cocaine abuse on ventral striatal D2/D3R availability through sleep duration (*b*=−0.221, 95% CI (−0.497, −0.054); Figure 1a). This represented an effect size *k*^2^=0.177, 95% Bca CI (0.051, 0.315), which is medium–large 0.09–0.25, respectively.^31^ Sleep also mediated the effect of cocaine abuse on D2/D3R in putamen (*b*=−0.163, 95% CI (−0.381, −0.043); *k*^2^=0.153, 95% Bca CI (0.047, 0.324)) and caudate (*b*=−0.171, 95% CI (−0.393, −0.056); *k*^2^=0.182 95% Bca CI (0.063, 0.387); see Figures 1b and c).

Mediation effects of sleep on striatal D2/D3R held when all the three models were corrected for potential covariates (that is, age, body mass index, gender, caffeine use, pack-years; see Table 2) to all the three models (betas including covariates: VS: *b*=−0.239, caudate: *b*=−0.190, putamen: *b*=−0.184; all 95% CIs≠0).

### Associations with behavioral measures in cocaine abusers

In cocaine abusers, there was a significant correlation between sleep duration and amount of cocaine last used in mg (*r*=−0.495, *P*=0.014) and in dollars (*r*=−0.543, *P*=0.006), but not with duration of cocaine use (*r*=0.141, *P*=0.51). Striatal D2/D3R availability was not associated with cocaine use measures (all *P*>0.204), providing further support that the decreased D2/D3R availability is mediated in part by sleep disruption.

Although sleep duration was not associated with scores for any of the Conners Adult ADHD Rating Scales (*P*>0.59), D2/D3R in caudate correlated with the hyperactivity scale at trend level in cocaine abusers (*r*=−0.400, *P*=0.053) but not in controls (*r*=−221, *P*=0.36).

## Discussion

Our findings confirm our hypothesis that sleep disturbances contribute to the reduction in striatal D2/D3R availability in cocaine abusers. They also replicate previous findings that cocaine abusers have shorter sleep durations^14^ and lower striatal D2/D3R availability compared with controls reviewed in refs 1, 2. Duration of sleep predicted D2/D3R availability in all striatal areas, also when correcting for group, which supports recent findings that a night of sleep deprivation decreases striatal D2/D3R availability in healthy volunteers^9, 10, 11^ and extends them to suggest that chronic sleep deprivation might also result in a downregulation of striatal D2/D3R. However prospective investigations are required to directly evaluate the effects of chronic sleep restriction on striatal D2R levels.

In our study, the relationship between cocaine abuse and reduced striatal D2/D3R availability was statistically mediated by sleep duration, as analyzed using a bootstrapping test of indirect effects.^29^ Mediation effects were present for all the striatal areas (that is, VS, caudate and putamen), also when controlling for potential covariates (that is, age, body mass index, gender, cigarette pack-years and caffeine use). As acute sleep deprivation has been found to reduce the striatal D2/D3R availability in the absence of substance abuse,^9, 10, 11^ the most parsimonious explanation of the current findings is that effects of cocaine on sleep duration is a causal mechanism linking cocaine abuse to D2/D3R availability. Such a mechanism may exist in addition to proposed direct mechanisms of chronic cocaine abuse on dopamine D2R and D3R receptor expression, for example, through G-protein-coupled reactions on adenylyl cyclase that may modulate the excitability of the neuron and D2/D3R gene expression levels.^32^ As both decreased sleep and D2/D3R reductions are present in many substance abusers, a causal relationship between sleep and D2/D3R may be a common mechanism underlying reductions in D2/D3R availability in the abuse of other substances. For example, the reductions in striatal D2/D3R in methamphetamine abusers^33^ might reflect methamphetamine-induced impairment of sleep.^34, 35^ Similarly, the reduced striatal D2/D3R availability in alcoholics^36^ could reflect their disturbed sleep^37^ and explain why both are indicators of alcohol relapse.^36, 38^ In contrast, active marijuana abusers who often use marijuana to promote sleep^39^ show no differences in D2/D3R availability compared with controls.^40, 41, 42, 43^ Future studies are necessary to investigate whether sleep also has a mediating role in reductions in D2/D3R availability in abusers of drugs other than cocaine, both in the abusing as well as in the withdrawal phases.

The findings of this study have implications for treatment of cocaine abuse. Reduced striatal D2/D3R availability in cocaine abusers has been associated with reduced function of prefrontal brain regions involved with executive function,^3^ increases in drug craving,^1^ relapse^4^ and worse clinical outcome after therapy.^6^ Improving and prolonging sleep in cocaine abusers may therefore be beneficial in increasing D2/D3R availability, which may further improve executive function and reduce the risk of relapse. For example, the wake-promoting drug modafinil makes cocaine-dependent individuals go to bed earlier and increases their slow-wave sleep,^17^ which should be tested to assess if it helps increase striatal D2/D3Rs. It was recently shown that improving REM sleep of rats in withdrawal from cocaine, decreased the animals' cocaine craving, which was associated with reduced accumulation of AMPA receptors in the nucleus accumbens of the VS.^16^ Interestingly, the acute administration of caffeine, which has wake-promoting properties, was recently shown to increase striatal D2/D3Rs^44, 45^ presumably through its antagonistic effects at the adenosine A2A receptors, which interfere with the internalization of D2/D3R.^46^ Thus, caffeine or A2A antagonist drugs might help prevent the decreases in striatal D2/D3R seen in cocaine abusers and future studies should evaluate this possibility. A prior study reported that caffeine use was lower in cocaine abusers than in controls, and regular caffeine use was related to less frequent cocaine use.^22^ However, in our study, we did not find between-group differences in caffeine intake. We also did not find evidence that self-reported daily caffeine intake was associated with striatal D2/D3R availability. Participants did not use caffeine at least 2 h before scanning, which eliminated confounds due to direct effects of caffeine on striatal D2/D3R availability.^44, 45^ Whether chronic rather than acute caffeine intake is associated with D2/D3R should be investigated in future studies, preferably in healthy controls controlling for confounding factors such as drug abuse and sleep disruptions.

The question remains as to how sleep reductions may influence D2/D3R. As sleep deprivation increases adenosine levels in the brain, which drive sleep pressure,^47^ we postulate a role of adenosine and A2A receptors in this process. Adenosine A2A receptors co-localize with D2/D3Rs in the striatum and antagonize them.^48^ Specifically, adenosine regulates the internalization of D2/D3Rs by facilitating β-arrestin-2 binding to the A2A-D2/D3R heteromer,^49^ thus downregulating D2/D3R availability in the extracellular membrane. It should be noted, however, that A2A receptor-induced internalization of the D2/D3R has not yet been demonstrated in the brain of animals *in vivo*. In rodents, the stimulation of adenosine receptors in the ventral striatum has been shown to reverse the expression of cocaine sensitization and cross-sensitization to D2/D3Rs.^50^ Future studies are needed to assess whether adenosine antagonists in cocaine abusers could help revert the downregulation of striatal D2/D3R^48^ and reduce cocaine intake. As adenosine receptor activation has also been shown to reduce the affinity of D2/D3R^51, 52^ and might influence dopamine release^53^ (but see refs 54, 55), other hypotheses should also be considered. That is, the association between decreased sleep durations and decreased [^11^C]raclopride binding in cocaine abusers may reflect decreases in dopamine release or receptor affinity. In a previous study, acute sleep deprivation did not increase dopamine release in a methylphenidate-challenged PET study in healthy volunteers along with microdialysis studies, hence suggesting downregulation of D2R and D3Rs.^9^ However, whether this also applies to chronic sleep deprivation is not yet known.

Reduced striatal D2/D3R after sleep deprivation can also impact other behaviors. For example, trait impulsivity has been associated with reduced D2/D3R^56^ as well as with disrupted sleep,^57^ which may enhance sensitivity for addiction.^58^ In our sample, Conners Adult ADHD Rating Scale impulsiveness was not associated with sleep or D2/D3R, though cocaine abusers showed higher scores on all the Conners Adult ADHD Rating Scale measures including inattention and hyperactivity. Nevertheless, we did show a weak association between D2/D3R in caudate and the hyperactivity scale at a trend level in cocaine abusers.

The main limitation of the study is that we relied on measures of self-report instead of using more thorough measures of sleep quality, such as polysomnography. Despite this, cocaine abusers have been shown to accurately estimate their total sleep duration: Morgan and colleagues showed that sleep durations measured with subjective reports (mean=6.6 h±1.5 s.d.) and polysomnography measures (mean=6.5 h±.73 s.d.) were strongly correlated (*r*=0.69) in 33 cocaine-dependent inpatients (Morgan *et al.*, unpublished data). Our self-reported sleep findings are also in line with previous polysomnography studies in cocaine abusers, that is, decreased sleep duration, increased sleep latency and more awake periods.^14^ Future studies using PET imaging and polysomnography are needed to assess the robustness of our findings. Another limitation is that mediation effects of sleep duration are statistical in nature, which makes it difficult to make causal statements about the effects. In fact, sleep disturbances in childhood have been shown to predict substance abuse in adolescence^59, 60^ and an alternative model in which reduced sleep, mediated by decreased striatal D2/D3R, predicts cocaine abuse could also find support. Despite the reduced number of sleep hours and disrupted pattern of sleep, the cocaine abusers in this study did not report feeling more tired upon awakening than controls. This is likely reflecting tolerance to the effects of chronic sleep deprivation, though we cannot rule out the possibility that it could also reflect differences in the number of hours of sleep that they need for proper function. Further, although cocaine, nicotine and caffeine use was well documented, we do not have data on recent alcohol or marijuana use, which may have influenced D2/3R availability or sleep patterns. We do know, however, that none of the subjects were diagnosed with present or past substance use disorders other than cocaine and nicotine dependence. Finally, our study could not assess whether chronic sleep disturbance in healthy controls would lead to downregulation of D2/D3R when cocaine is not acting as an intermediary.

In summary, in this study, we corroborate that both sleep and striatal D2/D3R availability are reduced in cocaine abusers, and document for, we believe, the first time that sleep reductions exert a statistically mediating role in the relationship between cocaine abuse and reduced striatal D2/D3R availability. As both reduced D2/D3R availability and sleep durations are predictors of relapse, these findings may call for strategies to improve sleep as part of the treatment interventions for cocaine abusers and potentially other substance abusers.

## Figures and Tables

**Table 1:** Clinical and demographic data for cocaine abusers and healthy controls

*Characteristic*	*Cocaine abusers,* N=*24*		*Healthy controls,* N=*21*		P*-value*
	*Mean*	*s.d.*	*Mean*	*s.d.*	
Age, years	44.78	4.88	42.18	5.49	0.10
Years of education	12.88	1.65	13.88	2.30	0.10
WAIS, *t*-score	51.22^a^	9.87	51.35^b^	10.18	0.97
BMI	25.37	4.41	26.10	2.21	0.48
Caffeine use (mg per day)	210.42	614.85	56.19	106.14	0.26
Cigarette pack-years	6.74	7.51	0.083	0.382	**<0.0001**
Last cocaine use (g)	3.60	3.22	—	—	—
Last cocaine use ($)	128.96	112.54	—	—	—
Cocaine use, years	19.50	6.53	—	—	—
Bed time	12.29 0014 h Range: 2200–0500 h	1.60 1 h 36 min	11.24 2314 h Range: 1900–0300 h	1.51 1 h 31 min	**0.029**
Wake up time	6.79 0636 h Range: 0400–1100 h	1.50 1 h 30 min	6.57 0634 h Range: 0100 –1000 h	1.78 1 h 47 min	0.66
Wake duration during night (h)	0.33 (19 min)	0.70 (42 min)	0	0	**0.029**
Sleep duration per night (h)	6.17 6 h 5 min Range: 3–8 h	1.61 1 h 37 min	7.43 7 h 26 min Range: 5–10 h	1.12 1 h 7 min	**0.004**
CAARS A inattention	45.83	16.30	33.05^c^	15.65	**0.013**
CAARS B hyperactivity	50.38	14.56	35.63^c^	16.62	**0.004**
CAARS C impulsivity	47.33	14.43	34.42^c^	15.89	**0.008**
BDI	11.17^a^	11.97	1.35^b^	2.98	**0.001**

Abbreviations: BDI, Beck's Depression Inventory; BMI, body mass index; CAARS, Conners Adult ADHD Rating Scale; WAIS, Wechsler adult intelligence scale.

a *N*=23.

b *N*=20.

c *N*=19. Bold significance levels are at *P*<0.05.

**Table 2:** Zero-order correlations between cocaine abuse, sleep duration and striatal D2/D3R, and potential covariates in the group of cocaine abusers and controls pooled together.

	*Cocaine abuse*	*Sleep duration*	*D2/D3R VS*	*D2/D3R putamen*	*D2/D3R caudate*	*Age*	*BMI*	*Gender*	*Caffeine use (mg per day)*
Cocaine abuse	1								
Sleep duration	−0.418^**^	1							
D2/D3R VS	−0.518^***^	0.546^***^	1						
D2/D3R putamen	−0.343^*^	0.454^**^	0.934^***^	1					
D2/D3R caudate	−0.182	0.449^**^	0.725^***^	0.804^***^	1				
Age	0.249	−0.273	−0.224	−0.177	−0.128	1			
BMI	−0.103	−0.072	−0.175	0.071	0.024	0.063	1		
Gender	0.071	−0.016	−0.204	−0.074	−0.144	0.007	−0.004	1	
Caffeine use (mg per day)	0.051	−0.050	−0.002	−0.116	−0.185	−0.006	−0.257	0.013	1
Cigarette pack-years	0.525^**^	−0.098	−0.264	−0.201	−0.134	0.092	−0.329*	0.128	0.255

Abbreviation: BMI, body mass index.

Cocaine abuse=cocaine abusers (1) versus healthy controls (0). Sleep duration=hours of sleep per night; D2/D3R is dopamine D2/D3R availability (Bmax/Kd) in caudate, putamen and ventral striatum (VS). Age in years. Gender=female (1) versus male (0). Caffeine use (mg per day)=estimated daily caffeine intake (coffee, tea, soda) in mg per day. Cigarette pack-years=(number of cigarettes per day × years of smoking)/20, with 20 being the size of a common pack of cigarettes.

Correlation coefficients in bold are significant at *P<0.05, **P<0.01, ***P<0.001.
